# Selected cannabis cultivars modulate glial activation: in vitro and in vivo studies

**DOI:** 10.1186/s42238-024-00232-0

**Published:** 2024-05-22

**Authors:** Sigal Fleisher-Berkovich, Nitzan Sharon, Yvonne Ventura, Valeria Feinshtein, Jonathan Gorelick, Nirit Bernstein, Shimon Ben-Shabat

**Affiliations:** 1 Department of Clinical Biochemistry and Pharmacology, Ben-Gurion University of the Negev, Beer-Sheva, Israel; 2 Institute of Soil Water and Environmental Sciences, Volcani Center, Rishon Lezion, Israel; 3 Eastern Regional Research and Development Center, Judea Center, 90100 Kiryat Arba, Israel

**Keywords:** Cannabis, Chemotype, Medical cannabis, Microglial activation, Nitric oxide, Multiple sclerosis, Neuroinflammation

## Abstract

**Introduction:**

Multiple sclerosis (MS) is a chronic autoimmune disease of the central nervous system characterized by neuroinflammation, demyelination and axonal loss. Cannabis, an immunomodulating agent, is known for its ability to treat MS effectively. However, due to variations in the profile of secondary metabolites, especially cannabinoids, among cannabis cultivars, the effectiveness of cannabis treatment can vary, with significant variability in the effects on different biological parameters. For screening available cultivars, cellular in vitro as well as pre-clinical in vivo assays, are required to evaluate the effectiveness of the wide range of chemical variability that exists in cannabis cultivars. This study evaluated comparatively three chemically diverse cannabis cultivars, CN2, CN4 and CN6, containing different ratios of phytocannabinoids, for their neuroinflammatory activity in MS model.

**Materials and methods:**

*In vitro* experiments were performed with lipopolysaccharide (LPS)-activated BV-2 microglia and primary glial cells to evaluate the effect of different cannabis cultivars on nitric oxide (NO) and inflammatory cytokines, as well as inducible nitric oxide synthase (iNOS) protein expression. An in vivo experiment using the experimental autoimmune encephalomyelitis (EAE) MS model was conducted using Myelin oligodendrocyte glycoprotein (MOG) as the activating peptide. The cannabis extracts of the cultivars CN2, CN4, CN6 or vehicle, were intraperitoneally injected with clinical scores given based on observed symptoms over the course of study. At the end of the experiment, the mice were sacrificed, and splenocyte cytokine secretion was measured using ELISA. Lumbar sections from the spinal cord of treated MS mice were evaluated for microglia, astrocytes and CD4^+^ cells.

**Results:**

Extracts of the CN2 cultivar contained tetrahydrocannabinolic acid (THCA) and tetrahydrocannabinol (THC) without cannabidiol (CBD), and a number of monoterpenes. CN4 contained cannabidiolic acid (CBDA) and tetrahydrocannabidiolic acid (THCA), with significant amounts of THC: CBD in a 1:1 ratio, as well as sesquiterpenes and some monoterpenes; and CN6 contained primarily CBDA and THCA, as well as THC and CBD in a 2:1 ratio, with some sesquiterpenes and no monoterpenes. All extracts were not cytotoxic in glial cells up to 50 µg/ml. Dose dependent inhibition of LPS-induced BV2 as well as primary microglial NO secretion confirmed the anti-inflammatory and anti-oxidative activity of the three cannabis cultivars. CN2 but not CN4 reduced both astrocytosis and microglial activation in lumbar sections of EAE mice. In contrast, CN4 but not CN2 significantly decreased the secretion of TNFα and Interferon γ (IFNγ) in primary splenocytes extracted from EAE mice.

**Conclusions:**

While both cannabis cultivars, CN2 and CN4, significantly reduced the severity of the clinical signs throughout the course of the study, they modulated different inflammatory mediators and pathways, probably due to differences in their phytocannabinoid composition. This demonstrates the differential potential of cannabis cultivars differing in chemotype to regulate neuroinflammation and their potential to treat MS.

## Introduction

Multiple sclerosis (MS) is a chronic autoimmune disease of the central nervous system (CNS) characterized by neuroinflammation, demyelination and axonal loss (Rahmanzadeh et al. 2018). A key aspect thought to modulate MS progression is the mechanism by which the brain responds to resident and infiltrating immune cells*.* Microglia are the most abundant immune cells in active MS lesions (Kuhlmann et al. 2017). Altered microglial activity and their interaction with resident astrocytes and CNS infiltrating cells is a critical determinant of the brain milieu in the disease (Cai et al. 2014).

Microglia play an important role in all stages of the development of MS. As part of the innate immune system, these cells respond to foreign/potentially dangerous stimuli (Jack et al. 2005). Activated microglia secrete inflammatory mediators such as cytokines, including tumor necrosis factor α (TNFα), prostaglandins, and free radicals such as Nitric Oxide (NO) (Cai et al. 2014). Microglial TNF-α molecules contribute to the demyelinating process (Wang et al. 2018). Interestingly, a TNFα- inducer, namely lipopolysaccharide (LPS), is linked by itself to neuroinflammation and microglial activation (Goshi et al. 2020) was found to be elevated in MS mice (Al-Ghezi et al. 2019). Exposure to LPS results in degeneration of myelin (Chu et al. 2019).

The cannabis plant, and specifically its phytocannabinoids such as cannabidiol (CBD) and tetrahydrocannabinol (THC), are known as immunomodulating agents (Almogi-Hazan & Or 2020). Current cannabis-based treatments for MS are based mainly on CBD and THC, even though there is evidence that other compounds in the plant also possess related therapeutic activity (Chiurchiu et al. 2018; Paes-Colli et al. 2022). Several studies have shown that the endocannabinoid system is affected by MS (Ranieri et al. 2016). Cumulative evidence in *ex vivo* trials has shown extensive changes in expression of the CB_1_ and CB_2_ receptors (CBRs) in several experimental models of MS and in patients in different clinical stages of the disease (Chiurchiu et al. 2018). These observations suggest a functional role of CBRs in the pathology of MS. It has encouraged preclinical studies aimed at modulating the activity of those receptors via phytocannabinoids or synthetic cannabinoid agonist/antagonists (Chiurchiu et al. 2018).

Experimental autoimmune encephalomyelitis (EAE) is an experimental autoimmune disease of the CNS that serves as an animal model for MS (Bjelobaba et al. 2018). For example, Baker et al. (2000), showed improvement in tremor and spasticity in EAE model mice when treated with plant cannabinoids, analogs, and CBRs agonists. By contrast, selective antagonists to these receptors inhibited clinical effects, indicating a possible involvement of the endocannabinoid system (Baker et al. 2000). In addition to treating symptoms, cannabinoids also contributed to neuronal protection through the CB_1_R and slowed down the neurodegenerative process (Pryce et al. 2003). Furthermore, cannabinoids reduced the progression of symptoms by suppressing immune system activity and encouraging re-myelination (Longoria et al. 2022).

In the chronic relapsing model of EAE (CREAE), increased levels of anandamide and 2-arachidonyl glycerol (2-AG), the two central endocannabinoids, were identified in the brain and spinal cord (Baker et al. 2001). By contrast, in the EAE model, only the levels of anandamide increased in the striatum (Centonze et al. 2007). In another model, the Theiler murine encephalomyelitis virus-induced demyelinating disease (TMEV-IDD), only 2-AG levels were increased in the spinal cord (Loria et al. 2008) (Cristino et al. 2020; Loria et al. 2008). Studies in mice with knockout CBRs have shown an increase in inflammation and symptoms in various MS models (Mestre et al. 2018). CB_1_R knockout mice showed an increase in inflammation in the TMEV-IDD model (Mestre et al. 2011) and neurodegeneration in the CREAE model (Pryce & Baker 2007). Knockout mice in the CB_2_R showed an increase in EAE severity and inflammation (Palazuelos et al. 2008). Mice without the endocannabinoid degrading enzyme, FAAH, develop EAE more easily (Rossi et al. 2011).

Synergistic interactions are known to occur between secondary metabolites in cannabis, including cannabinoids and terpenes (Comelli et al. 2008; Gallily et al. 2018). This phenomenon was termed the entourage effect, where the integrated impact of all compounds in the cannabis plant is greater than the sum of its parts (Ben-Shabat et al. 1998). The biological effect of cannabis cultivar extracts may therefore have higher efficacy compared to treatments with only individual cannabinoids (Comelli et al. 2008). The production of bioactive compounds in the cannabis plant is affected by genetics (Shiponi & Bernstein 2021b), location in the plant (Bernstein et al. 2019), and by cultivation conditions (Saloner and Bernstein 2021, 2022a; Danziger and Bernstein 2022). Since the secondary metabolite profile in cannabis varies between plant cultivars (Danziger & Bernstein 2021a; Saloner & Bernstein 2022b), so may their beneficial effects against neuroinflammation.

The present study therefore focused on comparing the effects of full spectrum extracts of inflorescences from chemically diverse cannabis cultivars against neuroinflammation. An *in vitro* model of neuroinflammation and in vivo model of EAE were used to compare extracts from three chemically diverse cannabis cultivars, grown under similar conditions against neuroinflammation. The acquired information may direct selection of specific cannabis strains for the treatment of neuroinflammation and multiple sclerosis.

## Materials and methods

### Plant cultivation: selected cannabis cultivars

Female plants from three cultivars of medical cannabis (CN2, CN4, CN6), 10 plants per cultivar, were produced from cuttings and grown simultaneously under uniform cultivation conditions in a controlled environment growing room as previously described (Shiponi & Bernstein 2021a, 2021b). In short, plants were cultivated in 4 L plastic pots in a perlite cultivation media (Agrekal, Habonim, Israel). During the vegetative growth phase, for 2 weeks, the plants were exposed to a 18/6 hrs light/dark photoperiod using Metal Halide bulbs (400 μmol m^-2^ s^-1^, Solis Tek Inc, Carson, California (Saloner & Bernstein 2020). For the remainder of the cultivation period, until flower maturation, the plants were grown under 12:12 light/dark photoperiod using High-Pressure Sodium bulbs (980 μmolm^-2^ s^-1^, Greenlab by Hydrogarden, Petah Tikva, Israel) (Saloner & Bernstein 2021). In the cultivation room, temperatures were kept at 26 and 24˚C day/night, respectively, and relative humidity at 44% and 60%, respectively. Irrigation was supplied via 1L h^-1^ discharge-regulated drippers (Netafim, Tel-Aviv, Israel) and mineral nutrients were supplied by fertigation, dissolved in the irrigation solution (Shiponi & Bernstein 2021a, 2021b).

### Drying

Inflorescences were harvested when about 30% of the trichome heads were of amber color, following the maturation stage acceptable for commercial harvesting. The inflorescences were immediately trimmed (wet-trimmed) to separate the protruding parts of the inflorescence leaves from the inflorescence. The trimmed inflorescences were then dried in an environmental-controlled chamber, in the dark, at 19˚C and 45% relative humidity until the plant material reached ~10% humidity, as is customary in the medical industry for material supplied to patients. Ten g of material sampled from 10 different primary inflorescences were ground thoroughly using a manual grinder, to create a homogenous mixture.

### Extraction and quantitative analysis of cannabinoids and terpenes in the cannabis cultivars

One gram of ground plant material was extracted in a shaker for 60 min in 30 ml of absolute ethanol and concentrated under reduced pressure. Dried extracts were heated for 30 min at 110°C to decarboxylate the cannabinoids from their natural acidic state to their neutral form. Samples were redissolved in ethanol to a final concentration of 10 mg/ml for further chemical and biological analysis. Cannabinoid quantification was performed as indicated (Aizpurua-Olaizola et al. 2014). A Waters Alliance 2695 Separation Module with a Waters 996 Photodiode Array Detector together with a Micromass Quattro Micro Triple Quadrupole Mass Spectrometer using a Phenomenex Kinetex C18 column (2.6 μm, 150 mm×3 mm i.d.) with guard column and a binary A/B gradient (solvent A: water with 0.1% formic acid, and solvent B: MeOH with 0.1% formic acid). Initial conditions were 65% B for 10 mins, raised to 95% B over the next 20 min, held at 95% B for 15 min, decreased to 65% B over the next 5 min, and held at 65% B for 10 min for re-equilibration of the system. The flow rate was 0.2 mL/min and the column temperature was 30 °C. Mass spectra acquisition was carried out in the ESI positive ionization mode under the following conditions: capillary voltage - 3.5 kV, cone voltage - 45 V, extractor voltage – 3 V, RF lens - 0.2 V, source temperature - 120 °C, desolvation temperature - 350 °C, nitrogen flow rate of 700 L/h for desolvation and 50 L/h cone gas. Quantification of the selected cannabinoids was performed using single ion monitoring (SIR) mode. Standard solutions of the selected cannabinoids (THC, THCA, CBD, CBDA, cannabigerol (CBG), cannabigerolic acid (CBGA), and cannabinol (CBN) were obtained from Merck (Rehovot, Israel). For each compound, serial dilutions were performed, and calibrations curves generated for concentrations from 0.5-100 µg/ml using SIR of the molecular ion [M+H].

For terpene analysis, 1 µl of the ethanolic extract was injected in a Hewlett Packard G 1800B GCD system with a HP-5971 gas chromatograph using a SPB-5 (30 m × 0.25 mm × 0.25 um) column with an electron ionization detector, using GCD Plus ChemStation (Hewlett Packard, Santa Clara, CA, USA) and a helium flow rate of 1 ml/min. Initial conditions consisted of an inlet temperature of 250 °C; detector temperature of 280 °C; and an initial temperature of 50 °C. The initial temperature was held for 5 min before increasing at a rate of 5 °C/min to a final temperature of 280 °C. Terpenes were identified based on retention time and mass spectra and quantified based on calibration curves generated from standards.

### Cell culture

#### BV2 murine microglial cells

Murine BV2 microglia were kindly provided by Professor Rosario Donato (Department of Experimental Medicine and Biochemical Sciences, University of Perugia, Italy) (Adami et al. 2004). Microglia were grown in RPMI-1640 medium with 10% fetal calf serum (FCS), streptomycin (100 µg/mL), penicillin (100 U/mL) and L-glutamine (4 mM) in 5% CO_2_ humidified air incubator at 37 °C. The culture medium was replaced twice a week.

For each experiment, cells were grown overnight on 6-well and 24-well plates at a concentration of 1×10^6^ and 3×10^5^ cells per well, respectively. Serum-free medium (SFM) was added to the cells 4 h before initiation of the experiment. Subsequently, microglia were treated with SFM containing 0.1% bovine serum albumin (BSA), 4-(2-hydroxyethyl)piperazine-1-ethane-sulfonic acid (HEPES) buffer (10 mM at pH 7.4), and 1% FCS in the presence of Lipopolysaccharides (LPS) (7 ng/ml) with or without extracts from 3 cultivars (CN2, CN4, CN6) at different concentrations (1, 5, 10, 20 µg/ml) for 22 h. All culture media were purchased from Biological Industries (Kibbutz Beit-Haemek, Israel). LPS from Escherichia coli O55:B5, was purchased from Sigma Aldrich (Rehovot, Israel).

#### Primary neonatal rat glial cells

According to well-established protocols, primary glial cell cultures were prepared from whole brains of 1-day old Wistar rats (Adami et al. 2004; Levant et al. 2006). Cells were grown in 24-well plates coated with poly-L-lysine (0.25 mg/mL) at 37 °C in 5% CO_2_. Dulbecco's modified Eagle's medium (4.5 mg glucose/mL), supplemented with 10% FCS, L-glutamine (2 mM), penicillin (100 U/mL), streptomycin (100 mg/mL) and insulin (0.2 U/mL) was used as the culture medium. As previously described (Filipovich-Rimon & Fleisher-Berkovich 2010), immunocytochemistry studies revealed that these cultures contain about 80% astrocytes and about 20% microglia. Before the experiment, cells were incubated in SFM for 4 h. LPS 500 ng/ml with or without extracts from 3 cultivars (CN2, CN4, CN6) (50 µg/ml) was added to SFM containing 0.1% BSA and HEPES (10 mM) for 22 h. At the end of each experiment, cells were collected with cold SFM (4 °C) and counted using the Z1 Coulter counter (Coulter Electronics, Miami, FL, USA).

#### Cell viability

Cells were seeded at a density of 1×10^4^ cells per well in 96-well plates and cultivated overnight in a complete RPMI-1640 medium (as described in 2.1.1). Increasing concentrations of CBG were given for 22 h following pre-incubation with SFM. Dimethylsulfoxide (DMSO) at a concentration of 0.026 % was used as a control. Actinomycin D, a transcription inhibitor at a concentration of 1 μM, was applied to inhibit cell proliferation. (2,3-Bis-(2-Methoxy-4-Nitro-5-Sulfophenyl)-2*H*-Tetrazolium-5-Carboxanilide) (XTT) reagent [2,3-Bis-(2- methoxy 4-nitro-5-sulfophenyl)-2H-tetrazolium5-carboxanilide] was mixed with the activation reagent, N-methyl dibenzopyrazine methyl sulfate, in a ratio of 50:1 according to the manufacture's protocol (Biological Industries, Kibbutz Beit-Haemek, Israel). Subsequently, XTT solution mix was added to each well in a 1:2 ratio, and plates were incubated at 37 °C for an additional 1 h. Absorbance was measured at 450 nm against a reference wavelength at 650 nm using a microplate reader (Model 680, Bio-Rad, Hercules, CA, USA). Cell viability was calculated using the following formula: [A_450_–A_650_] of test cells × 100/[A_450_-A_650_] of control cells.

#### Determination of NO levels (Griess reaction)

As an indicator for NO release, nitrite levels in culture media were determined by an established assay using Griess reagent (Sigma Aldrich, Rehovot, Israel). A standard curve of sodium nitrite was used. 100 µL of culture medium and Griess reagent were mixed in a 96-well plate and incubated at room temperature for 15 min in the dark. Then, absorbance at 540 nm was measured using a microplate reader (Model 680, Bio-Rad, Hercules, CA, USA). Cells were harvested with cold SFM (4°C) and counted using the Z1 Coulter counter (Coulter Electronics, Miami, FL, USA).

#### Determination of TNFα levels (ELISA)

TNFα levels in the culture media were determined by enzyme-linked immunosorbent assay (ELISA) (BD Biosciences, San Diego, CA) according to the manufacturer's protocol.

#### Western blot analysis

Proteins were separated on polyacrylamide-SDS (7.5%) gels and transferred to nitrocellulose membranes. After blocking with 4% BSA for 90 min at room temperature, membranes were incubated overnight at 4 °C with a specific rabbit anti-iNOS antibody (1:500, Cayman Chemicals, USA). Upon washing, the blots were incubated for 90 min at room temperature in the corresponding conjugated donkey anti-rabbit antibody (1:10,000, GE Healthcare, Buckinghamshire, UK). The position of the individual protein was detected using enhanced chemiluminescence (ECL) solution followed by exposure to the ChemiDocTM XRS+ (Bio-Rad Laboratories, Hercules, CA, USA) image system. Band intensity analysis was performed using a computerized image analysis system (ImageJ software, version 1.40C, NIH). Protein quantity was normalized to β-actin protein level measurements using mouse monoclonal anti-β-Actin−Peroxidase antibody (1:20,000, Sigma Aldrich, Rehovot, Israel).

#### Active MOG-induced EAE model

Female C57BL/6 mice, eight weeks old (~20 g) (Envigo, Jerusalem, Israel), were grown for one week and then immunized with myelin oligodendrocyte glycoprotein (MOG) [peptide 35–55] (AnaSpec, Fremont, CA, USA). Under anesthesia with isoflurane, each mouse was injected subcutaneously (SC) into two sites on the back, adjacent to each of the hind limbs (total volume 200 μL), with 200 μg MOG emulsified with a mixture of 200 μg/mL killed Mycobacterium tuberculosis H37RA (Difco, Detroit, MI, USA) in complete Freund's adjuvant (BD Biosciences, USA). After that, each animal was injected i.p with reconstituted pertussis toxin (ENCO scientific services, Petach Tikvah, Israel) (400 ng/mL), which was repeated two days after the initial immunization.

Control EAE mice received a vehicle solution composed of Tween-20: ethanol: saline at a ratio of 1:1:8. Wet samples were dissolved in a vehicle solution. IP administration was used for seven consecutive days from day 7 to 14 post-immunization (p.i.). The mice were evaluated for a neurological score as follows: 0 normal; 0.5 mild ataxia of the hind limb; 1 decreased tail tone; 1.5 righting reflex within 3 s; 2 righting reflex between 4 and 7 s; 2.5 righting reflex between 7 and 10 s; 3 hind limbs paralysis or complete loss of righting reflex; 4 front and hind limbs paralysis; 4.5 moribund state; 5 death. At the end of the experiment, mice were anesthetized, and cardiac perfusion was performed. The spinal columns were fixed in 4% formaldehyde at 4 °C overnight and cryoprotected in 20% sucrose for 48 h at 4 °C. Then, spinal cords were dissected and mounted in OCT (Scigen Scientific Gardena, CA, USA), snap-frozen at -40 °C, and finally stored at - 80°C for further procedure. Free-floating sections (30 µm thick) were blocked for 1 h in blocking buffer [Antibody diluent (GBI Labs, Mukilteo, WA, USA) with 0.5% Triton], immunostained overnight at 4 °C with antibodies diluted in antibody diluent with 0.15% Triton. The following antibodies were used: monoclonal anti-glial fibrillary acidic protein (GFAP, clone GA5; 1:400) [MAB360, Millipore], rat anti-CD4 (clone RM4-5; 1:50) [BD Pharmingen] and rabbit anti-Iba1 (1:1,000) [FUJIFILM Wako Pure Chemical Corporation]. The next morning, sections were washed three times with 0.05 % Tween 20 in PBS, then incubated for 1 h at room temperature with a fluorescent-conjugated secondary anti-rat, anti-mouse, or anti-rabbit antibody (1:200, Alexa Fluor 647; Jackson). Subsequently, DAPI was applied for nuclear staining. Sections were mounted on slides with Immu-Mount (Thermo Scientific, MI, USA). Images were obtained using the Olympus FluoView FV1000 confocal microscope (Olympus, Hamburg, Germany) at a 1024×1024 pixel resolution.

#### Primary splenocytes

Splenocytes were isolated 15 days p.i by mashing the splenic tissue through a 2 μm filter and washing with PBS+EDTA 1mM. The collected fluid was once again passed through the strainer into a test tube and cells were centrifuged at 4 °C. The cell pellet was incubated with red blood cells (RBC) lysis buffer Ethylenediaminetetraacetic acid(EDTA) 10mM + NaHCO3 0.1M + NH4Cl 1.5M in DDW, 2 mL buffer/a spleen) for 10 min at room temperature. The RBC-lysed splenocytes were washed with PBS+EDTA 1mM and resuspended in the culture medium (DMEM) supplemented with 10% bovine calf serum, 1% penicillin/streptomycin, Sodium pyruvate (1%), HEPES buffer (10 mM at pH 7.4), 1% non-essential amino acids, and 0.02% 2-mercaptoethanol. Cells were seeded in a 96-well plate in U-shaped wells and incubated at 37 °C for 48 h, at which time the supernatants were removed for analysis by enzyme-linked immunosorbent assay (ELISA).

#### Imaging analysis

Glial fibrillary protein (GFAP), ionized calcium binding adapter molecule 1 (Iba1) and cluster of differentiation 4 (CD4) staining were quantified in lumbar sections from the spinal cord of each mouse using ImageJ software (version 1.40C, NIH) with the threshold function. An intensity threshold was set to mark only those areas showing significant staining. Identical laser‐scanning parameters were used for all samples. The indicated proteins' averaged positive‐stained areas were calculated separately for each treated group and plotted as integrated density.

### Statistical analysis

Experimental data are presented as means ±SEM. For significance assessment between groups, a one-way analysis of variance (ANOVA) and post hoc multiple comparison test (Tukey-Kramer Multiple Comparison Test) were performed using Prism version 5.00 for Windows, GraphPad Software, (San Diego, California, USA). Statistical significance was considered at *p*< 0.05.

## Results

### Extraction and analysis of phytocannabinoids and terpenes from selected cannabis cultivars

Based on data from preliminary viability *in vitro* tests with six cannabis cultivars, we selected three non-cytotoxic cannabis cultivars (CN2, CN4 and CN6) for further evaluation.

Concentrations of phytocannabinoids and terpenes in extracts from inflorescences of the cultivars used in the study are presented in Table 1, and Table 2, respectively.

**Table 1:** Concentrations of phytocannabinoids in extracts from the three cannabis cultivars evaluated in the study (CN2, CN4, CN6). Cannabigerolic acid (CBGA), Cannabigerol (CBG), Cannabidiolic acid (CBDA), Canna bidiol (CBD), Tetra-Hydrocannabinolic acid (THCA), Tetrahydrocannabinol (THC), Cannabinol (CBN)

**Cultivar**	**THC/CBD ratio**	**Concentrations of cannabinoids in the cannabis extracts (mg/ml)**						
		**CBGA**	**CBG**	**CBDA**	**CBD**	**THCA**	**THC**	**CBN**
CN2	High THC	n.d.	0.16	n.d.	n.d.	4.63	5.84	0.1
CN4	Balanced	n.d.	0.04	3.83	1.46	0.94	2.06	0.09
CN6	Balanced	n.d.	0.12	3.49	1.63	1.38	3.96	0.1

^a^*n.d.* Not detected

**Table 2:** Concentrations of terpenes in extracts from the three cannabis cultivars (CN2, CN4, CN6)

**Concentrations of terpenoids in the cannabis extracts (mg/ml)**			
**Terpenoid**	**Cultivars**		
	**CN2**	**CN4**	**CN6**
α-pinene	0.01	n.d.	n.d.
β-pinene	0.053	n.d.	n.d.
β-myrcene	0.127	0.01	n.d.
d-limonene	0.017	0.02	n.d.
3-carene	0.005	n.d.	n.d.
fenchyl alcohol	n.d.	0.05	n.d.
caryophyllene	0.317	0.17	0.272
α-guaiene	n.d.	0.02	n.d.
α-caryophyllene	0.106	0.06	0.114
caryophyllene oxide	0.046	0.02	n.d.
guaiol	n.d.	n.d.	0.084
β-maaliene	n.d.	n.d.	0.141
eudesmol	n.d.	n.d.	0.219
α-bisabolol	0.053	0.09	0.129

*n.d.* Not detected (lower than the detection limit)

### NO production in BV2 cells

**Fig. 1:**
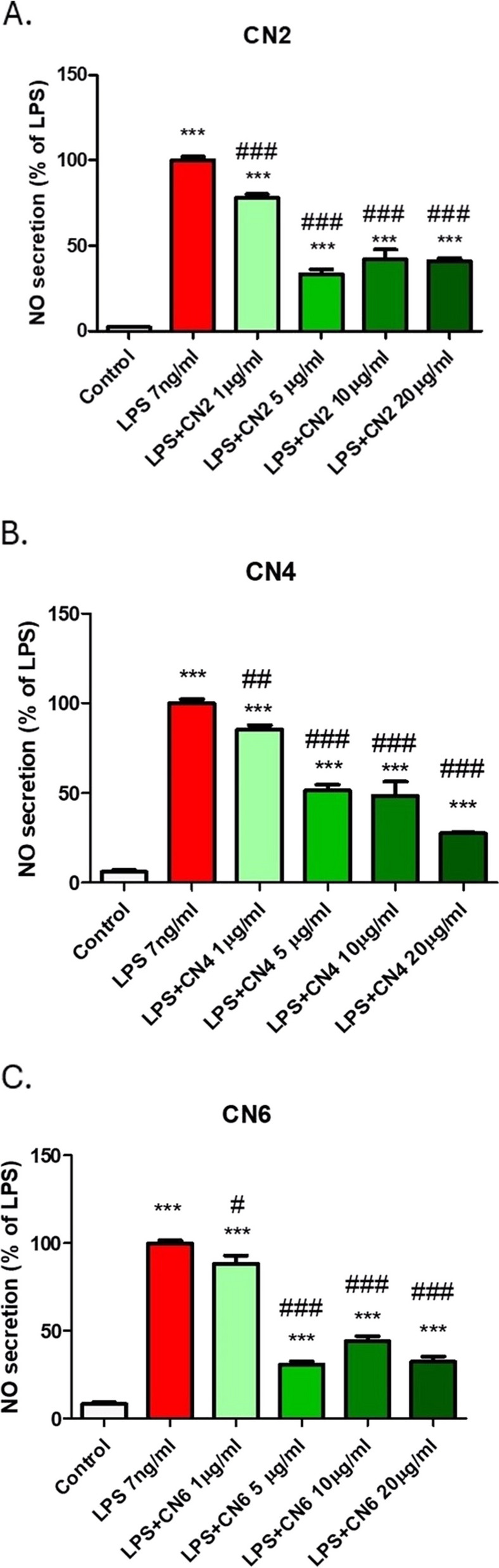
Extracts of the cultivars CN2, CN4, CN6 reduced NO secretion in BV-2 cells. BV2 cells were incubated for 24 h with LPS (7 ng/ml) and extracts from the selected cannabis cultivars (CN2 (**A**), CN4 (**B**), CN6 (**C**)) at different concentrations (1, 5, 10, 20 µg/ml). The amount of NO in the medium was determined, and the cells were counted at the end of the experiment. The results (means ±SEM, *n*=18) are representative results from three independent experiments. Statistical significance was determined by one-way ANOVA and Tukey-Kramer Multiple Comparison Post Test. *** *p* <0.001 vs., control, ### - *p* <0.001 vs., LPS

CN2, CN4, and CN6 extracts at a 50 µg/ml concentration were safe to use on BV2 microglia based on viability tests. NO measurement performed with increasing concentrations of the extracts (1, 5, 10, and 20 µg/ml) in BV2 cells activated by LPS, revealed a decrease in NO levels (*** *p* <0.001 vs., control, ### -* p* <0.001 vs., LPS) (Fig. 1).

### iNOS expression in LPS-activated BV2 cells

**Fig. 2:**
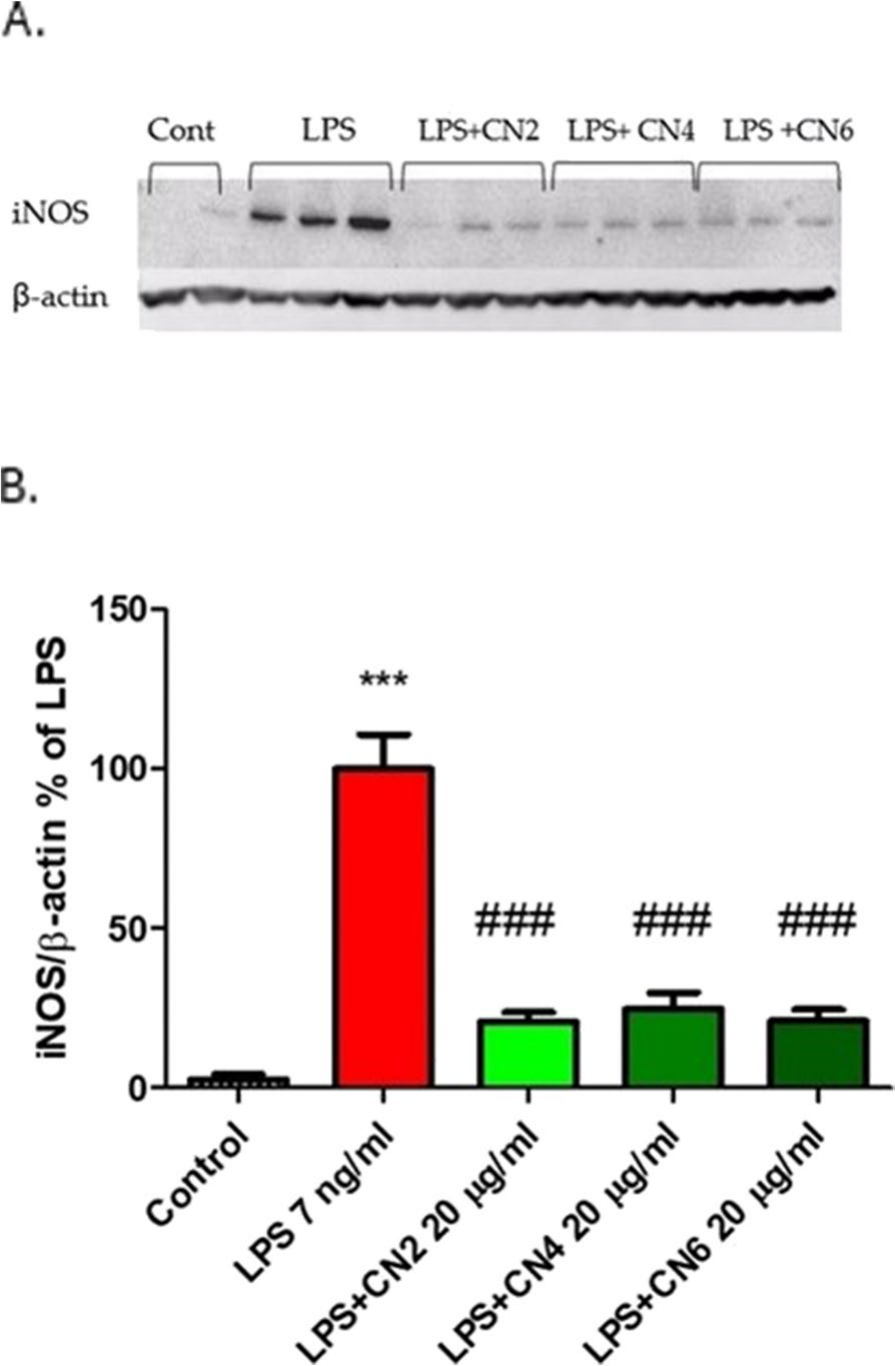
Extracts of the cannabis cultivars CN2, CN4, and CN6 lowered iNOS protein expression in LPS-activated BV2 cells. BV2 cells were incubated for 24 h with LPS (7 ng/ml) and extracts from selected cannabis cultivars (20 µg/ml). At the end of the experiment, SDS PAGE and Western blot analysis were performed using antibodies against iNOS and β-actin. In A, the bands represent two independent experiments. In B, the results are mean±SEM of all experiments, (*n*=6). Statistical significance was determined by one-way ANOVA and Tukey-Kramer Multiple Comparison Post Test. *** - *p* <0.001 vs. control, ### - *p* <0.001 vs. LPS (*n*=6). *** - *p* <0.001 vs. control, ### - *p* <0.001 vs. LPS

The effect of extracts from the cannabis cultivars, CN2, CN4, and CN6 on iNOS protein expression in LPS-activated BV-2 cells is presented in Fig. 2. The three cultivars significantly reduced iNOS expression (***p <0.001 vs. control*. p* <0.001. ### *p* <0.001 vs LPS), but without significant differences between cultivars.

#### NO production in primary rat glial cell

**Fig. 3:**
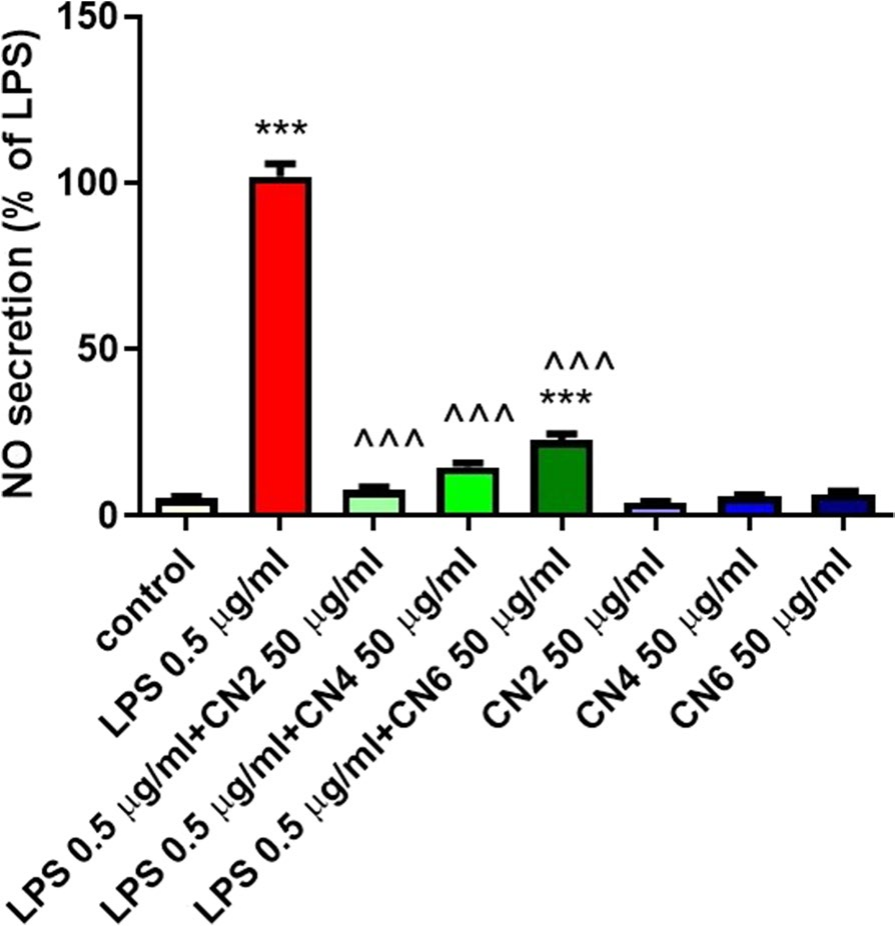
Extract of the cannabis cultivars (CN2, CN4, CN6) significantly reduced NO secretion in primary glial cells. Primary glial cells were incubated for 24 h with or without LPS (0.5µg/ml) and extracts from the cannabis cultivars CN2, CN4, or CN6 (50µg/ml). The amount of NO in the medium was determined, and cells were counted at the end of the experiment. The results are representative results from three independent experiments., 3 independent experiments were performed, *n*=18. *** - *p* <0.001 vs. control, ^^^ - *p* <0.001 vs. LPS. Statistical significance was determined by one-way ANOVA and Tukey-Kramer Multiple Comparison Post Test

The effect of the extracts on NO secretion in primary glial cells activated by LPS was evaluated at non-cytotoxic concentrations (up to 50 µg/ml). In all the cultivars tested, a decrease in NO levels was observed with CN2 and CN4 almost completely mitigating the effects of LPS (Fig. 3).

### Cannabis cultivars in in vivo EAE model

**Fig. 4:**
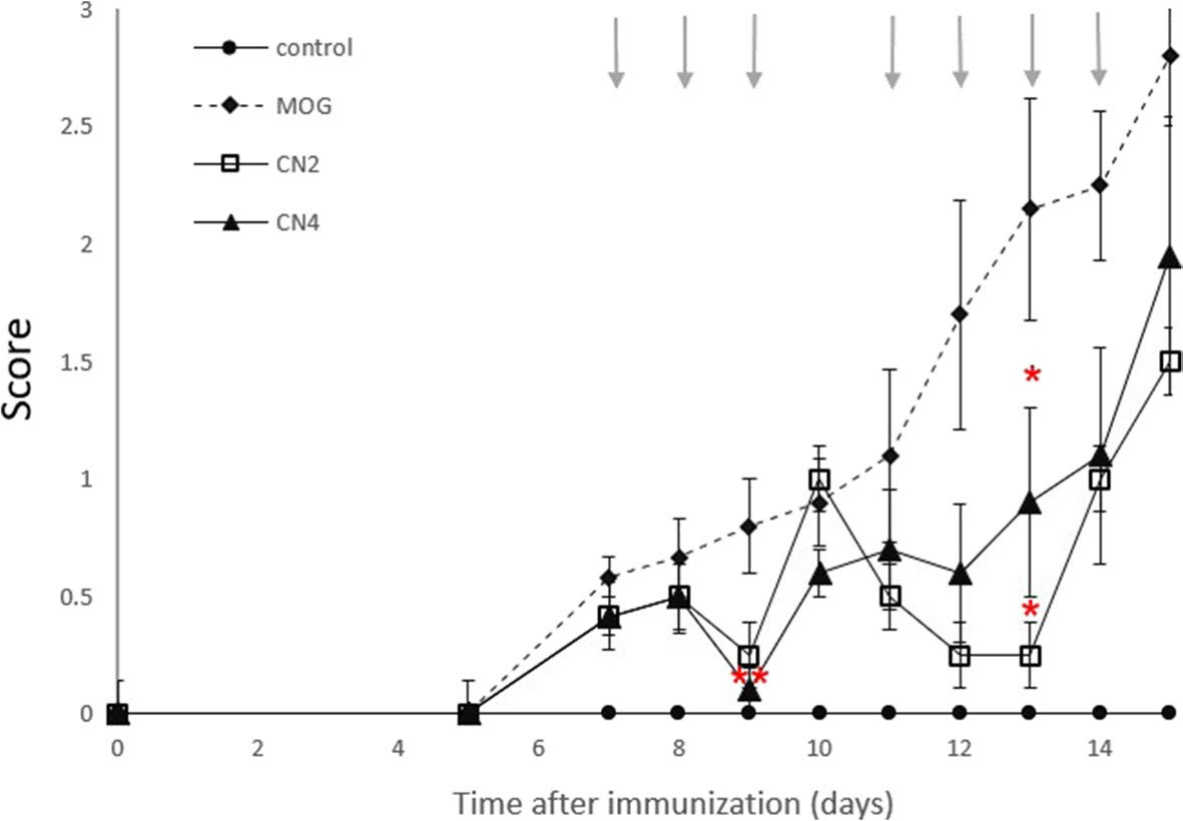
Extracts from the selected cannabis cultivars ameliorated EAE symptoms in MOG immunized mice. Mice treated with MOG35-55 in the absence or presence of CN2 or CN4 extract once daily (days 7, 8: dose 10 mg/kg, days 9, 10, 12-14: 5 mg/kg). Presented results are mean ± SEM. Negative control group without vaccine and treatment. ** *p* <0.01, * *p* <0.05 vs. MOG. Statistical significance was determined by one-way ANOVA and Tukey-Kramer Multiple Comparison Post Test (*n* = 5)

The EAE animal model of MS was used to evaluate the effects of cannabis cultivars on disease scores (Fig. 4). Clinical scores are one of the validated behavioral methods used to evaluate the symptoms of EAE in mice. Following induction of EAE with MOG35-55, the symptoms of EAE mice with and without treatment with extracts from the cannabis cultivars (CN2 or CN4) were compared by clinical score testing. Symptoms started on day six and became increasingly severe throughout the course of the experiment. Both cannabis cultivars tested, lowered the severity of the scores compared to the non-treated EAE mice over the course of the experiment, with the difference becoming statistically significant on days 9 and 13 post-treatment. Interestingly, while both cannabis cultivars lowered the mean score to close to 0 on day 9, only CN2 produced a similar almost complete reduction of symptoms on day 13. CN4 treated mice also displayed a significant reduction, of over 50%, at the termination of the study. No effect was observed with CN6 (data not shown).

#### Effect of cannabis cultivars on TNF-α and IFNγ secretion in primary spleen cells

**Fig. 5:**
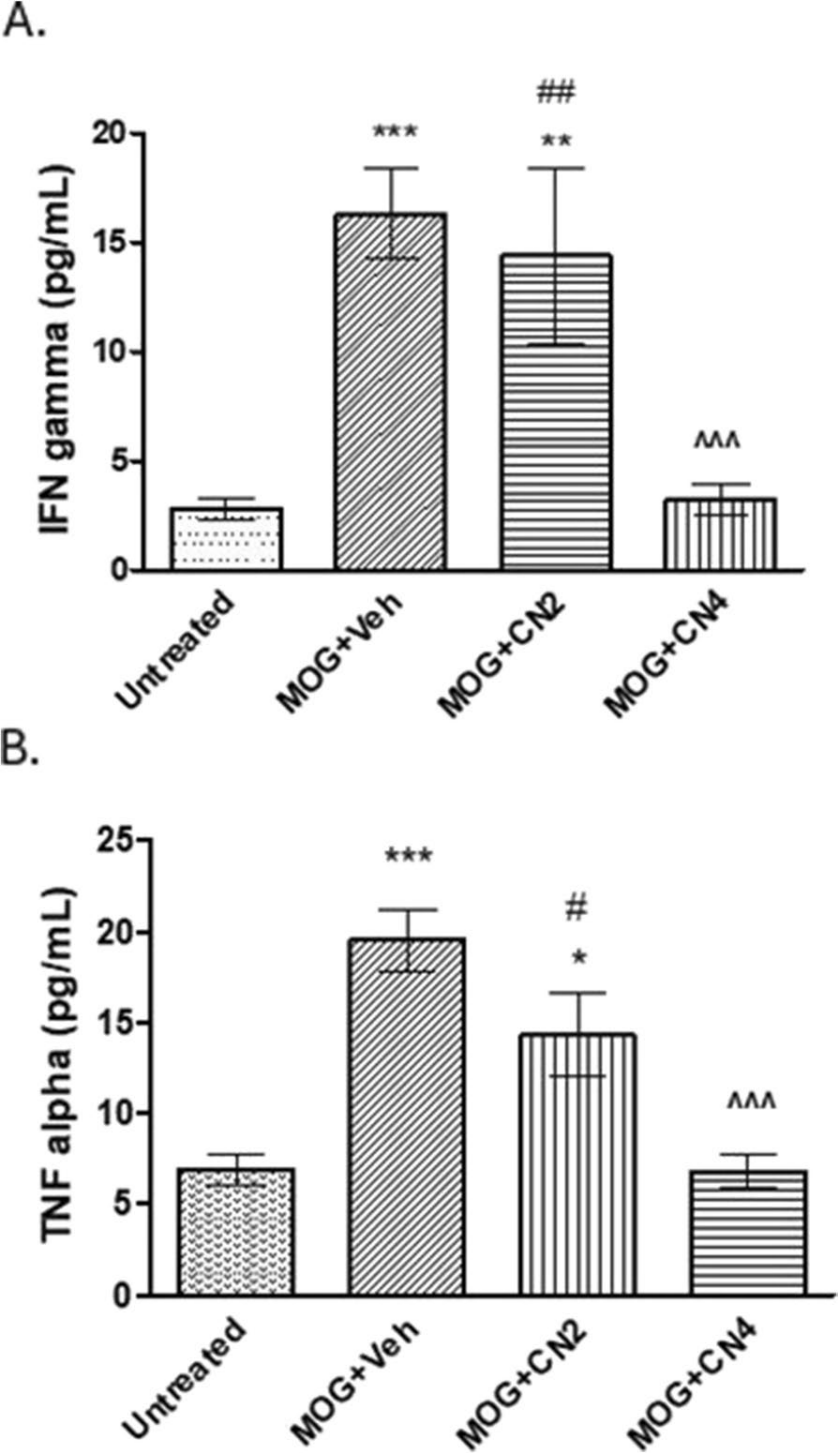
TNFα and IFNγ secretion in splenocytes from EAE mice treated with extracts of 2 cannabis cultivars. At the completion of the EAE studies, splenocytes were harvested from mice untreated or treated with MOG35-55 (MOG) injected with vehicle (Veh) CN2 or CN4 cannabis cultivars. The medium was collected after 48 hours and tested for IFNγ (**A**) and TNFα levels (**B**) by ELISA. Presented results are means±SEM. Statistical significance was determined by one-way ANOVA and Tukey-Kramer Multiple Comparison Post Test - *** *p* <0.001 vs. control, ** *p* < 0.01 vs. control, * *p* <0.05 vs. control, ^^^* p* <0.001 vs. MOG, ^ *p* <0.05 vs. MOG, ## *p* <0.001 vs. CN4 # *p* <0.05 vs. CN4, #. *n* = 5-15

At the end of the animal studies, spleens were harvested to evaluate the effects of cannabis treatment on cytokine secretion. As expected, in the MOG group, there was a significant increase in the secretion of TNFα and IFNγ compared to the unvaccinated control group (Fig. 5). In the CN4 treated group, both TNFα and IFNγ levels were significantly lowered, by >70%. Treatment with CN2 did not significantly reduce the levels of TNFα or IFNγ.

### Activation of microglia in the spinal cord of CN2 and CN4 treated mice

**Fig. 6:**
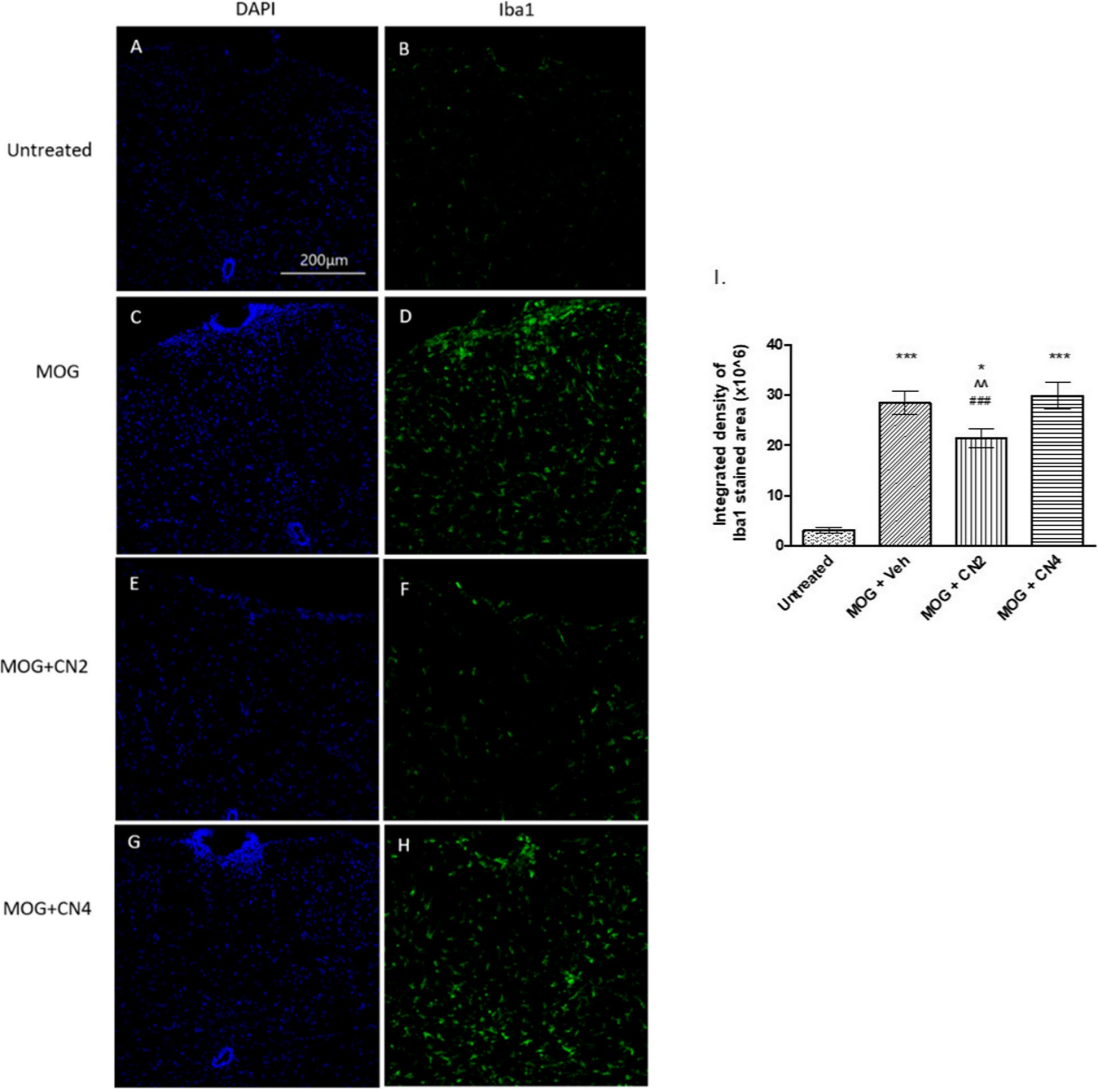
Microglial activation in the spinal cord of cannabis-treated mice. Female C57BL / 6 mice treated with MOG35-55 were treated with vehicle, CN2, or CN4. Spinal cords were removed and cut horizontally. The sections were stained by immunohistochemistry using antibodies against activated microglia (green), and the cell nuclei were stained by DAPI (blue) and examined using a confocal microscope (X20 magnification). The images (**A**-**H**) are representative images of 2 independent experiments, with 5 mice in each treatment group. In I, the presented results are mean ±SEM of the color intensity to the area of the Iba1 results (B,D,F,H), n ≥ 20 *** - *p* <0.001 vs. control, * *p* <0.05 vs. control , ^^ *p* <0.01 vs. MOG, ### *p* <0.001 vs. CN4

Treatment with cannabis cultivars affected Iba-1 activation in microglia in the lumbar region of the spinal cord in MOG treated mice (Fig. 6). As expected in this EAE model, vaccination with MOG significantly increased microglial activation compared to the unvaccinated control. Treatment with CN2 significantly reduced microglial activation by around 50% while CN4 produced a slight, not significant increase.

### Activation of astrocytes in the spinal cords of CN2 and CN4 treated EAE mice

**Fig. 7:**
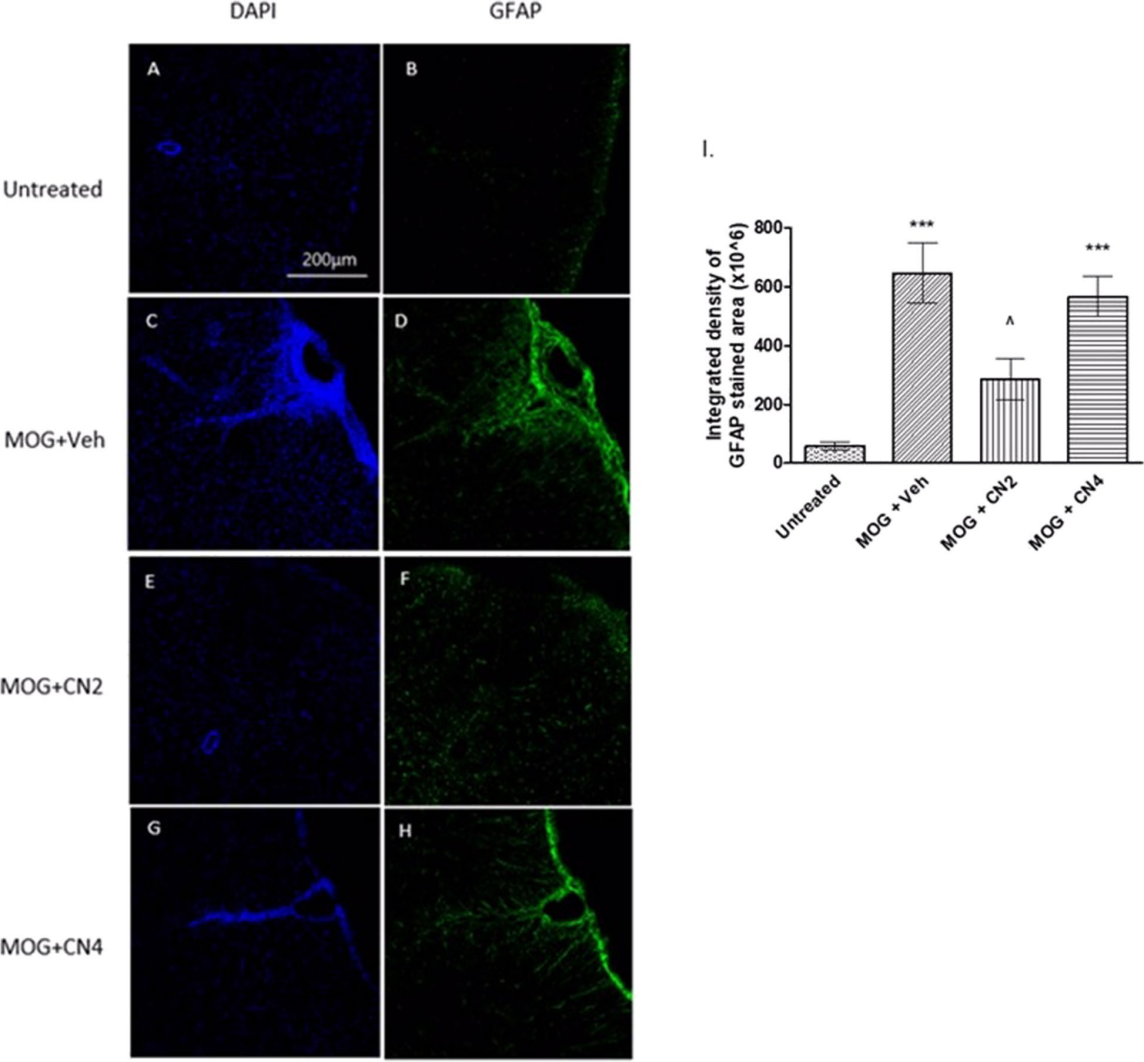
Astrocyte activation in the spinal cord of cannabis-treated mice. MOG35-55-given female C57BL/6 mice were treated with vehicle, CN2, or CN4. Spinal cords were removed and cut horizontally. Sections were stained using antibodies against GFAP (green), and the cell nuclei were stained with DAPI (blue) and examined using a confocal microscope (X20 magnification). The images (A-H) are representative images of 2 independent experiments, with 5 mice in each group. In I, the graphical result is presented as the mean ±SEM of the color intensity to the area, n ≥20. *** - *p* <0.001 vs. control, ^ *p* <0.05 vs. MOG

The effect of the CN2 or CN4 cultivars on astrocyte activation in the lumbar region of the spinal cord of treated mice is presented in Fig. 7. Astrocytes were stained (green) by the GFAP astrocyte marker. While there was almost no staining of astrocytes in the control group, MOG significantly increased astrocytosis. Treatment with CN2 produced a 60% reduction in astrocytosis, while CN4 did not produce any significant change, similar to the results observed regarding microglial activation.

### Infiltration of CD4^+^ T cells into the spinal cord of CN2 and CN4 treated EAE mice

**Fig. 8:**
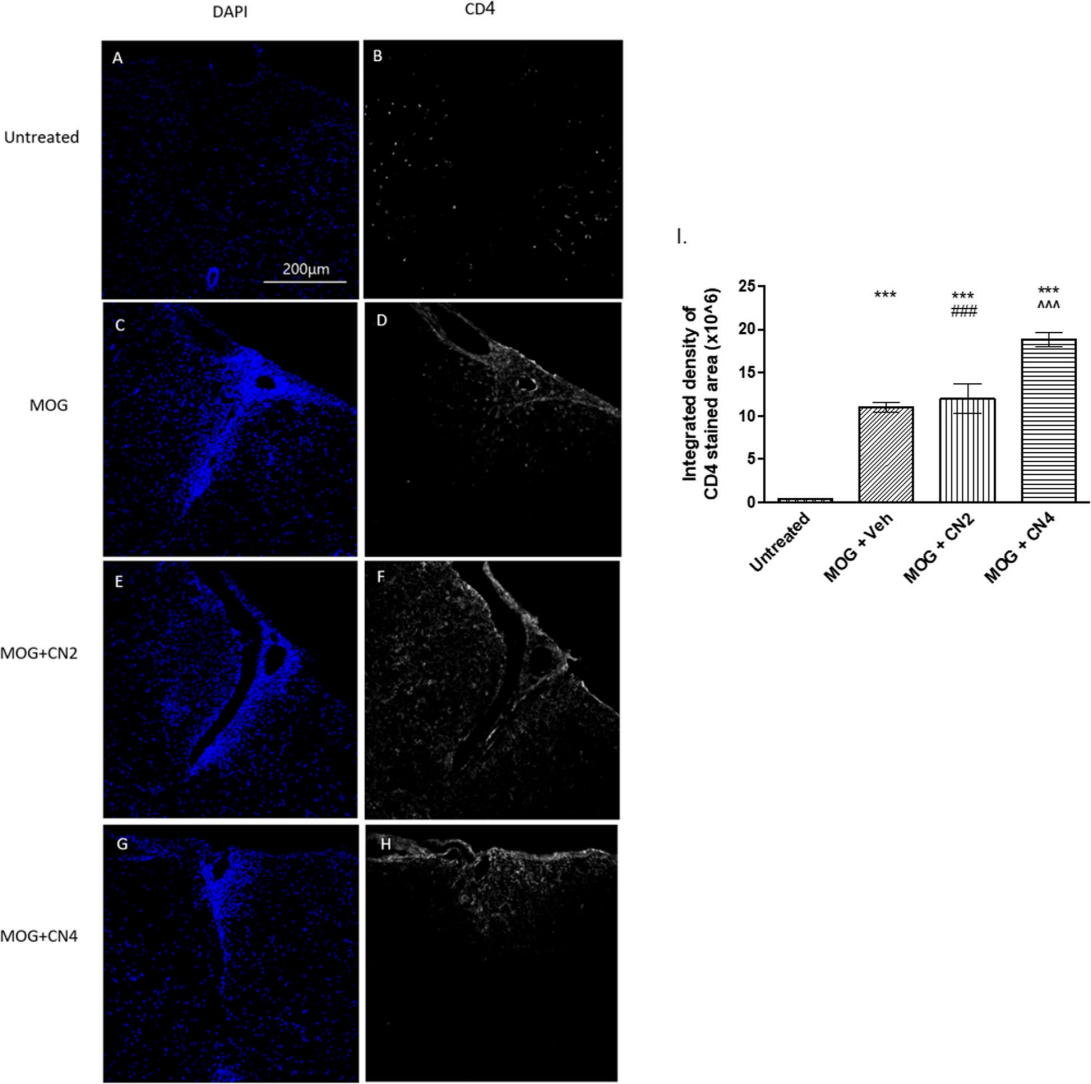
Infiltration of CD4^+^ T cells into the spinal cord of cannabis-treated mice. MOG35-55-given female C57BL/6 mice were treated with vehicle, CN2, or CN4. Spinal cords were removed and cut horizontally. Sections were stained using antibodies against CD4^+^ T cells (gray), and the cell nuclei were stained with DAPI (blue) and examined using a confocal microscope (X20 magnification). The images (A-H) are representative images of 2 independent experiments, with 5 mice in each group. In I, the graphical result is presented as the mean ±SEM of the color intensity to the area, n ≥10. *** - *p* <0.001 vs. control, ^^^*p* <0.001 vs. MOG, ### *p* <0.001 vs. CN4

CD4^+^ cells infiltration was evaluated by analyzing the total intensity of anti-CD4 immunofluorescence in the lumbar region of the spinal cord of CN2 and CN4 treated mice. MOG-immunization enhanced CD4^+^ T cells infiltration to the spinal cord compared to the control group. A significant increasing of CD4^+^ T cells infiltrating was identified in spinal cords of CN4-treated mice, while CN2 did not produce any significant change, compared to the MOG+Veh group (Fig 8)

## Discussion

MS is an autoimmune disease of the nervous system that affects myelin. MS progression is accompanied by glial inflammation. Microglia dually regulate inflammation. Sometimes these cells induce an inflammatory response. However, in other cases, microglia induce disease improvement by clearance of cell debris and myelin (Guerrero & Sicotte 2020). Astrocytes are also a main component of MS plaques, positioned to induce inflammation by cytokines such as TNFα and reactive oxygen species (ROS) like NO, but they may also reduce disease impairment by providing metabolic support to axons (Williams et al. 2007). While glial NO itself is less active, it can react with a superoxide anion to form peroxynitrite (ONOO−), one of the most harmful ROS/reactive nitrogen species (ROS/RNS) (Encinas et al. 2005; Islam 2017). Peroxynitrite plays an essential role in the pathology of demyelinating diseases, such as MS, mediating the process of demyelination, axonal loss and neurodegeneration (Lan et al. 2018).

In this study, we examined effects of extracts from three cannabis cultivars on modulation of the glial inflammatory response. We used cultivars which are approved in Israel for medical use to alleviate symptoms of MS. Female plants from the three cultivars were produced simultaneously from cuttings, and grown under uniform conditions. The phytocannabinoid profile of cannabis flowers is known to depend on genetics, along with their growing conditions (Eichhorn Bilodeau et al. 2019; Danziger and Bernstein 2021b; Shiponi and Bernstein 2021a). Therefore, standardization of the growing conditions for different cannabis cultivars is essential for comparative analysis of the cultivars’ therapeutic activity (Gorelick & Bernstein., 2017), as well as for obtaining consistency among various treatments.

After testing the cytotoxicity of six cultivars in BV2 microglial cells (data not shown), the non-toxic strains, CN2, CN4 and CN6, were selected for further study. The CN2 cultivar is a High-THC variety, with the extract containing high levels of THCA and THC with low levels of CBG. Extracts from CN4 and CB6 contained similarly high concentrations of both CBD and CBDA. The CN6 extract contained even higher levels of THC and THCA, and the CN4 extract contained lower but still significant levels of THC and THCA. A variety of terpenes were identified in the extracts, specifically monoterpenes, including α-and β-pinene, and myrcene in CN2. Fewer monoterpenes but more sesquiterpenes were found in CN4, and no monoterpenes but some sesquiterpenes in CN6.

This difference in chemical composition may explain the different effects produced by the different cannabis cultivars. Interestingly, a major difference between the extracts of the cultivars was in the levels of the acid forms of the cannabinoids. While considered for years as pharmacologically inactive due to their lack of direct affinity to the classical cannabinoid receptors, the acidic cannabinoids were more recently revealed to be of pharmacological interest. More than a decade ago, CBDA was first identified as an anti-inflammatory cannabinoid, inhibiting cyclooxygenase-2 (COX-2) (Takeda et al. 2008). Later, THCA was recognized as a peroxisome proliferator-activated receptor-γ (PPAR-γ) agonist with anti-inflammatory activity in vitro and in vivo (Nadal et al. 2017). In addition, the therapeutic effects of terpenes have been well documented with α-pinene shown to possess anti-inflammatory and neuroprotective activity (Salehi et al. 2019). The high levels of THCA (over the range of extract concentrations tested, 1-50 µg/ml, the concentration of THCA ranged between 1.3-65 µM) as well as monoterpenes such as α-pinene present in CN2 may at least partially explain the observed activity including reduced clinical score and specifically decreased astrocytosis and microglial activation. The high levels of CBD (over the range of extract concentrations tested, 1-50 µg/ml, the concentration of CBD ranged between 0.5-25 µM) and CBDA (over the range of extract concentrations tested, 1-50 µg/ml, the concentration of CBDA ranged between 1-53 µM) with known anti-inflammatory activity may explain the reduction in TNFα and INFγ secretion observed in CN4.

LPS which is linked to neuroinflammation and microglial activation (Chu et al. 2019), was found to be elevated in the brains of EAE mice and to leads to myelin degeneration (Al-Ghezi et al. 2019). We demonstrated the anti-inflammatory and anti-oxidative effects of three cannabis cultivars, revealed by the reduction of LPS-induced NO, and iNOS in BV2 (Figs. 1, 2 and 3) and in primary microglial cultures (Fig. 4). Next, we investigated the therapeutic potential of i.p administration of CN2, CN4 and CN6 upon disease onset, on neurological scores of EAE female mice. Female EAE mice were chosen to be studied since autoimmune diseases are more prevalent in females than males, a discrepancy also found in animal models (Ortona et al. 2016). Increased spinal cord lesions and demyelination are found more in female EAE mice vs. males (Wiedrick et al. 2021).

Treatment of the mice was initiated upon disease onset. The CN2 and CN4 cultivars reduced significantly the severity of the clinical symptoms throughout the experiment (Fig. 4), while CN6 showed no improvement of clinical score of mice (data not shown). CN2 but not CN4 reduced both astrocytosis, microglial activation in lumbar sections of EAE mice. EAE is characterized by the migration of activated T cells from the periphery to the CNS (Rangachari & Kuchroo 2013). In the spleen, T cells promote the synthesis of many cytokines in favor of stimulating inflammation (Van den Eertwegh et al. 1992). In the current study, primary splenocytes were extracted from control and EAE mice. CN4 significantly decreased the secretion of TNFα and IFNγ by 80% and 74%, respectively, while CN2 showed no beneficial effect on the above cytokine secretion. The EAE trial with MOG on CB_1_-deficient mice, and a second induction of the spleen cells extracted from them, offers a dual effect of the CB_1_ receptor on the immune response, which varies depending on the cell types activated. Furthermore, the treatment of EAE mice with rimonabant (CB_1_R antagonist) increased IFNγ production in spleen cells that were reactivated with MOG and other pro-inflammatory cytokines. These studies suggest the possibility of CB_1_R involvement in cytokine secretion (Nichols & Kaplan 2021).

Here, CN2 significantly reduced the MOG-induced astrocytosis and microgliosis in lumbar sections of spinal cords in EAE mice. CN4 treatment did not affect the activation of microglia and astrocytes compared to the untreated group. However, CD4 cell migration from the periphery was increased compared to the other groups. Interestingly, specific transcripts amplified following MOG treatment were maintained at high levels in the presence of CBD in the spleen cells of C57BL/6 mice. These include chemokines (Ccl3, Ccl4, Cxcl10) that promote cell migration (Kozela et al. 2016). This report is in accord with our results, which show that CN4 (which contains CBD) increased the migration of CD4 cells. CD4 cells, through secretion of neurotrophic factors such as the brain derived neurotrophic factor (BDNF) and the neuronal growth factor (NGF), promote neuronal survival (Wang & Tian 2021). CD4 cells can reduce MHC-type II expression on microglial cells, regulate phagocytosis by microglial cells, and inhibit the proliferation of oligonucleotides (Bradl et al. 2005).

The two cannabis cultivars (CN2 and CN4) reduced significantly the severity of the clinical signs along the course of the disease, compared to the untreated group. It is possible that these cultivars, probably due to differences in their chemical composition, modulate different inflammatory mediators' synthesis and related pathways that eventually lead to significant effects on the inflammatory environment.

## Conclusion

This study demonstrates the potential of cannabis cultivars for regulating inflammatory mediators, and the role of immune cells in vitro and in vivo. Cannabinoids, as well as additional substances identified in the cannabis cultivars, such as terpenes, may contribute to the anti-inflammatory properties observed (Goncalves et al. 2020). Since the chemical profiles of the cultivars’ extracts were primarily characterized, it is possible in the future to test combinations of individual cannabinoids’ combinations, in comparison with the total extracts. Of course, the multifaceted roles of cannabinoids and their combinations in glial cells suggests that different combinations may have a wide array of effects on neuroinflammation.

## Data Availability

All data are included in the manuscript.
